# Effect and Molecular Mechanisms of Jiedu Recipe on Hypoxia-Induced Angiogenesis after Transcatheter Arterial Chemoembolization in Hepatocellular Carcinoma

**DOI:** 10.1155/2021/6529376

**Published:** 2021-01-12

**Authors:** Wanfu Lin, Huan Wang, Maofeng Zhong, Shasha Yu, Shasha Zhao, Shufang Liang, Juan Du, Binbin Cheng, Wei Gu, Changquan Ling

**Affiliations:** ^1^Department of Traditional Chinese Medicine, Changhai Hospital, Second Military Medical University, Shanghai 200433, China; ^2^Department of Gastroenterology, Shanghai Municipal Hospital of Traditional Chinese Medicine Affiliated to Shanghai University of TCM, Shanghai 201900, China

## Abstract

Transcatheter arterial chemoembolization (TACE) is one of the effective treatment methods for hepatocellular carcinoma (HCC) in middle and late phases. However, TACE-induced hypoxia may promote the angiogenesis and section of some cytokines, such as IL-8, and, thereby, lead to tumor metastasis. Therefore, we investigated the effect of Jiedu Recipe (JR), which has been demonstrated as an effective Traditional Chinese Medicine (TCM) recipe on HCC, on TACE-induced cytokines upregulation and hypoxia-induced angiogenesis. A total of 88 hepatocellular carcinoma (HCC) patients treated with TACE were enrolled and divided into a JR group or control group. TACE induced significant increases of neutrophil lymphocyte ratio (NLR), platelet lymphocyte ratio (PLR), IL-1*β*, IL-2R, IL-6, and IL-8. JR treatment significantly inhibited the elevation of IL-8 compared with control. *In vitro*, JR significantly inhibited the hypoxia-induced overexpression of IL-8, HIF-1*α*, and VEGF mRNA in Huh 7 cells. ELISA assay demonstrated the effect of JR on IL-8 expression. Both hypoxia and IL-8 may promote angiogenesis which was suppressed by JR. Western blot showed that IL-8 upregulated the expression of phosphorylation of AKT, ERK, NF-*κ*B, and VEGFR, which were inhibited by JR. On the other hand, effects of IL-8 on the increase of p-AKT and p-ERK were also blocked by LY294002 and U0126, respectively. In conclusion, our results indicated that JR may inhibit hypoxia-induced angiogenesis through suppressing IL-8/HIF-1*α*/PI3K and MAPK/ERK pathways after TACE in HCC patients.

## 1. Introduction

Hepatocellular carcinoma (HCC) accounts for >80% of liver cancer, which occurs worldwide with 782,500 new cases annually [1, 2]. HCC surveillance and early detection may increase the opportunity of curative treatments such as surgical resection, local ablation, or liver transplantation [3]. However, most HCC patients are diagnosed middle and late phases due to the aggressive growth and late symptom presentation. For middle-phase HCC patients, transcatheter arterial chemoembolization (TACE) is an effective treatment option. In TACE, embolic material, usually the lipiodol, is injected to block the tumor-feeding artery through the hepatic artery. However, clinical practice and previous studies showed that complete embolization of tumor is usually impossible [4, 5]. In the tumor microenvironment, incomplete embolization results in relative hypoxia and, thereby, elevates the expression of hypoxia-inducible factor-1*α* (HIF-1*α*) [6, 7].

In HCC, the role of HIF-1*α* has been widely investigated. Overexpression of HIF-1*α* induces the upregulation of angiogenic growth factors such as VEGF and promotes formation of tube by vascular endothelial cells and vasculogenic mimicry by tumor cells [8–10]. In addition, HIF-1*α* also involves in the induction of some inflammatory cytokines, such as IL-8 [11, 12], which may also promote the expression of VEGF independently [13]. Although multikinase inhibitor sorafenib may target the VEGF receptors (VEGFRs), platelet-derived growth factor receptor (PDGFR), etc., which are key factors of angiogenesis, its combination with TACE did not improve time-to-tumor progression in a clinically meaningful manner when compared with TACE alone [14]. Another multikinase inhibitor lenvatinib showed the same result [15]. Therefore, neoangiogenic mechanisms after TACE is complex, and novel treatments are urgently needed.

Traditional Chinese medicine, mainly used in China for thousands of years, has been demonstrated by many studies that it may retard HCC progression, improve quality of life in HCC patients, and prevent HCC occurrence either alone or in combination with other conventional therapies, including TACE [16–18]. Jiedu Recipe (JR), which contains *pseudobulbus cremastrae seu pleiones*, *valvate actinidia root*, *salvia chinensis*, and *endothelium corneum gigeriae galli*, may prolong the survival of patients with advanced HCC [19]. Our retrospective study also indicated that TACE combined with JR may improve the prognosis and prolong survival of patients with unresectable HCC when compared to the patients treated with TACE alone [20].

However, the mechanisms beyond JR have not been fully investigated. Therefore, the aim of the present study was to explore the effect of JR on HCC, on TACE-induced cytokines upregulation, and hypoxia-induced angiogenesis.

## 2. Materials and Methods

### 2.1. Study Design

We conducted a prospective cohort study of 88 patients diagnosed with liver cancer and treated with TACE at Changhai Hospital, Second Military Medical University (Shanghai, China), from February 2018 through August 2018. Eligible participants for this study were over 18 years of age; diagnosed with liver cancer and treated with TACE; Child–Pugh grade A or B; and did not receive any herbal or systemic treatment for 2 weeks prior to the start of the trial. Patients who received treatment that may affect the effect of the trial; who participated in other clinical trials; and who had other serious diseases that may affect treatments in the trial were excluded.

Enrolled patients were assigned to the control group or JR group randomly after providing written informed consent. All patients received TACE treatment in Changhai Hospital, and supportive care such as liver-protecting treatment and antiemetics was permitted in the both groups. Besides, patients in the JR group may take JR at a dose of 4.5 g orally twice daily for 2 months (JR was prepared with herbs of *pseudobulbus cremastrae seu pleiones*, *valvate actinidia root*, *salvia chinensis*, and *endothelium corneum gigeriae galli* in a ratio of 2.5 : 2.5 :  1 :  1 by Jiangyin Tianjiang Pharmaceutical Company, Jiangsu, China).

Concentration of serum IL-1*β*, IL-6, IL-8, TNF-*α*, platelet lymphocyte ratio (PLR), and neutrophil lymphocyte ratio (NLR) were detected before and 3 days and 2 months after TACE.

The protocols of the present trial were approved by the First Affliated Changhai Hospital of Second Military Medical University Ethics Committee (Shanghai, China) (Approval No: CHEC2018-074).

### 2.2. Preparation of the JR

The JR lyophilized powder was produced by Shanghai Winherb Medical Technology Co., Ltd. (Shanghai, China) through extracting herbs of *pseudobulbus cremastrae seu pleiones*, *valvate actinidia root*, *salvia chinensis*, and *endothelium corneum gigeriae galli* in a ratio of 2.5 : 2.5 :  1 :  1 (w/w). In detail, the abovementioned dried and pulverized herbs were mixed together and were extracted with 85% ethanol by heat under reflux. This step was repeated twice, and the extracts were mixed. Then, the mixture was filtrated and further concentrated under vacuum at 50°C, productions from which were freeze-dried (with cold trap temperature −56°C) to obtained the lyophilized powder and stored at 4°C before used. For quality control, high-performance liquid chromatography assay was performed to determine the fingerprinting of JR in the School of Pharmacy, Second Military Medical University. In the *in vitro* assays, JR was dissolved in the cell culture medium and filtered twice with a 0.22 *μ*m filter.

### 2.3. Cell Lines

The human HCC cell line Huh 7 and human immortalized endothelial cells, EA.hy 926, were obtained from the Cell Bank of the Chinese Academy of Sciences Committee Type Culture Collection (Shanghai, China). The cells were cultured in high glucose Dulbecco's modified Eagle's medium (DMEM) (Thermo, Shanghai, China) supplemented with 10% fetal bovine serum (FBS, Gibco Life Technologies, Carlsbad, CA, USA), 100 U/mL penicillin, and 0.1 mg/mL streptomycin (Hyclone, Life Sciences, USA). The cells were cultured in a humidified atmosphere of 95% normal air and 5% CO_2_ at 37°C. Cells were passaged after cell density reaching 80% confluence and used in the experiment as in their logarithmic growth phase.

### 2.4. Cell Viability MTT Assay

To examine the effect of JR on cell growth, EA.hy 926 cells were seeded into 96-well plates with 5 × 10^3^/well and cultured overnight. Then, cells were treated with varying concentrations of the JR (0.2, 0.4, 0.8, 1.6 mg/mL) for 24 or 48 h, and 20 *μ*L MTT solution (5 mg/mL) was added to each well and incubated at 37°C for an additional 4 h. After carefully removing the culture medium, 150 *μ*L dimethyl sulfoxide was added to each well to dissolve the crystals. A multiskan spectrum microplate reader (Thermo Fisher Scientific, Waltham, MA, USA) was used to measure absorbance of the converted dye in living cells at a wavelength of 570 nm.

### 2.5. Capillary-Like Tube Formation Assay

The capillary-like tube formation assay was used to evaluate angiogenesis as described in the previous studies with modification [21]. Briefly, Matrigel (BD Biosciences, USA) was thawed at 4°C overnight before it was administered into the cold 96-well plate with 50 *μ*L gel/well and incubated at 37°C for one hour. Then, EA.hy 926 cells suspended in serum-free DMEM with a concentration of 5 × 10^5^/mL were plated to the plate with 100 *μ*L/well. Cells were treated with cobalt chloride (CoCl_2_), which was used to induce hypoxia-like condition, or human IL-8 protein (R&D Systems, USA) or JR. After 16 h incubation at 37°C, tube formation ability of the cells was visualized under the inverted phase contrast microscope (Leica, Germany) and evaluated by counting the number of junctions, total segments length, and mean mesh size with the open source software Image J (version 1.51).

### 2.6. Real-Time Reverse Transcription-Polymerase Chain Reaction Assay

Total RNA was isolated from Huh 7 cells by using Trizol reagent (Invitrogen, USA) as described previously [22]. The first-strand cDNA was synthesized using the PrimeScript RT reagent Kit (Takara, Japan) according to the manufacturer's procedures. qPCR was performed in a CFX96 Real-Time PCR system (Bio-Rad, CA, USA) using a commercial SYBR Green PCR Master Mix (TOYOBO, Osaka, Japan). The relative gene expression was measured and normalized to *β*-actin by the 2^−∆∆Ct^ method. The primers used in this experiment were as follows: HIF-1*α*, forward: 5′-TTC CCG ACT AGG CCC ATT C-3′, reverse: 5′-CAG GTA TTC AAG GTC CCA TTT CA-3′; VEGF, forward: 5′-GCC TCG GGC TTG TCA CAT TTT-3′, reverse: 5′-CCC TGA TGA GAT CGA GTA CAT CT-3′; IL-8, forward: 5′-TCT TGG CAG CCT TCC TGA TT-3′, reverse: 5′-TGG TCC ACT CTC AAT CAC TCT CAG T-3′; And *β*-actin, forward: 5′-AGC GGG AAA TCG TGC GTG-3′, reverse: 5′-CAG GGT ACA TGG TGG TGC C-3′.

### 2.7. Enzyme-Linked Immunosorbent Assay (ELISA)

Protein levels of IL-8 in cell culture supernatants were measured with ELISA assay. Huh 7 cells were treated with CoCl_2_ with or without 0.2, 0.4, and 0.8 mg/mL JR for 24 h. Cell culture media were then collected and centrifuged at 1500 × g for 20 min to obtain the supernatants. ELISA assay was carried out with Human IL-8 ELISA kits (R&D Systems, USA) to determine the IL-8 levels in the obtained supernatants according to the manufacturer's instructions.

### 2.8. Western Blot Assay

Total protein was extracted from treated EA.hy 926 or Huh 7 cells with cell lysis buffer (Cell Signaling Technology, USA) containing protease and phosphatase inhibitors, and protein quantification was carried out with a BCA protein assay kit (Thermo, USA). Equivalent protein content was then separated by electrophoresis and transferred to the PVDF membranes. The membranes were first blocked for 1 h with 5% skim milk and then incubated with primary antibodies overnight. After incubated with horseradish peroxidase-conjugated secondary antibodies for 2 h at room temperature, target protein blots were detected by enhanced chemiluminescence reagents (Thermo, USA).

### 2.9. Statistical Analysis

SPSS 19.0 software was used for all statistical analyses, and data were presented as mean ± standard deviation. For the clinical data, Student's *t*-test or chi-square test was used to compare the difference between the groups and Fisher's exact test was used for analysis of categorical variables. For the *in vitro* assay, one-way ANOVA followed by Tukey's test was used for the analysis. All results were considered statistically significant when *P* < 0.05.

## 3. Results

### 3.1. Patient Characteristics

A total of 88 patients meeting the study criteria were enrolled with 47 in the control group and 41 in the JR group. The baseline data for the patients in the 2 groups were comparable in terms of age, gender, Child–Pugh score, performance status, and BCLC stage (Table 1).

### 3.2. JR Inhibited IL-8 Elevation after TACE

**Figure 1:**
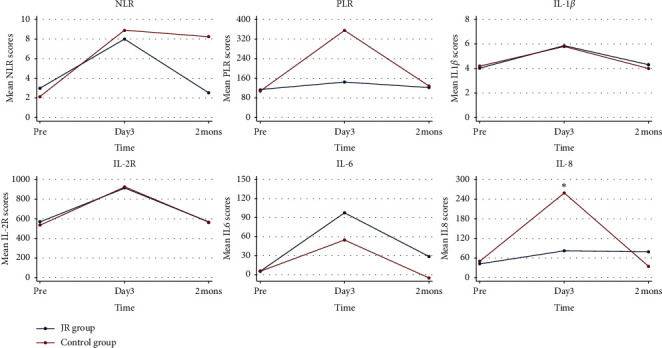
Changes of inflammatory cytokines before and after TACE. NLR: neutrophil lymphocyte ratio; PLR: platelet lymphocyte ratio; IL: interleukin; IL-2R: interleukin-2 receptor. ^*∗*^*P* < 0.05, compared with the control group.

The changes of inflammatory cytokines before and after TACE were examined to determine the target of JR. As shown in Figure 1, NLR, PLR, and all the detected cytokines, including IL-1*β*, IL-2R, IL-6, and IL-8, were significantly upregulated on the 3rd day in the control group after TACE compared to those before TACE (*P* < 0.01). On the 3rd day after TACE, the levels of PLR and IL-8 were lower in the JR group than the control group, indicating JR administration may suppress TACE-induced upregulation of PLR and IL-8 (Figure 1).

### 3.3. Effect of JR on Endothelial Cells Proliferation

**Figure 2:**
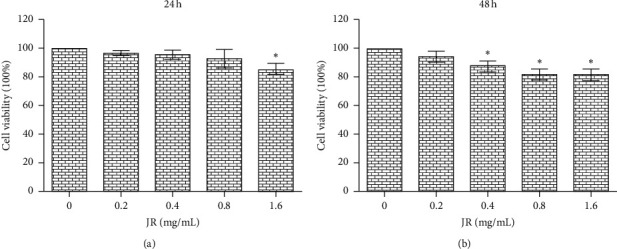
Effect of JR on EA.hy 926 cells' proliferation. MTT  assay was used to determine the cell proliferation. EA.hy 926 cells were treated with 0.2, 0.4, 0.8, and 1.6 mg/mL JR for 24 h (a) and 48 h (b). Data are expressed as means ± S.D. ^*∗*^*P* < 0.01 vs. control group.

To explore whether JR may inhibit angiogenesis *in vitro*, MTT assay was firstly conducted to evaluate the effects of JR (0, 0.2, 0.4, 0.8, and 1.6 mg/ml) on the proliferation of human immortalized endothelial cells EA.hy 926. Data presented in Figure 2(a) showed that, after treatment for 24 h, JR at the concentration of 1.6 mg/mL significantly inhibited the proliferation of EA.hy 926 cells compared with control (*P* < 0.05) (Figure 2(a)). When treated for 48 h, JR at the concentration of 0.4–1.6 mg/mL significantly suppressed the endothelial cells proliferation (Figure 2(b)). However, the maximum inhibitory rate was only 18.29% for JR.

### 3.4. Effect of JR on Capillary-Like Tube Formation under Hypoxic Condition

**Figure 3:**
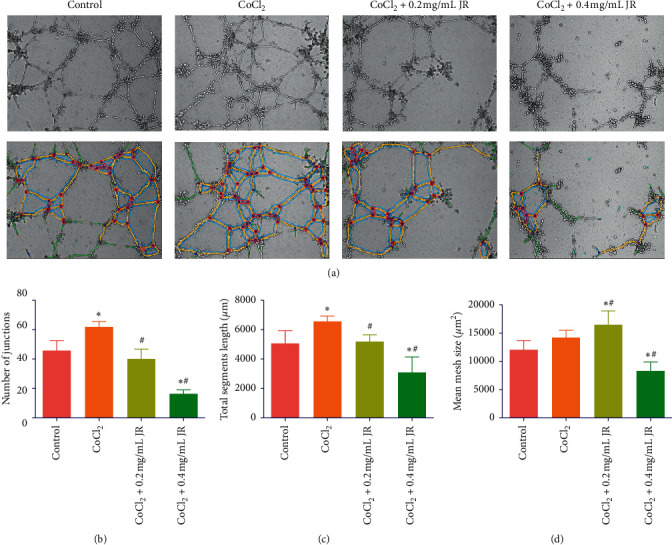
Effect of JR on capillary-like tube formation under hypoxic condition. EA.hy 926 cells were treated with CoCl_2_ and JR for 16 h. (a) Inverted phase-contrast microscopy images of capillary-like tube formation and tubule branches marked out by Image J software (magnification of 100x). ((b)–(d)) Number of junctions, total segments length, and mean mesh size of the tube, respectively. Data are expressed as means ± S.D. ^*∗*^*P* < 0.05 vs. control group and ^#^*P* < 0.05 vs. CoCl_2_ group.

Effect of JR on the angiogenesis was evaluated by capillary-like tube formation assay *in vitro*. Hypoxia induced by CoCl_2_ significantly stimulated the formation of capillary-like tube, which was significantly attenuated by JR treatment (Figure 3(a)). In detail, we used the open source software Image J to mark out the tubule branches (Figure 3(a)) and calculated the number of junctions, total segments length, and mean mesh size to assess the tube formation ability quantitatively (Figures 3(b)–3(d)). CoCl_2_ treatment significantly increased the number of junctions and total segments length compared with the control group. Also, JR significantly decreased the number of junctions and total segments length at both 0.2 and 0.4 mg/ml (Figures 3(b) and 3(c)).

### 3.5. Effect of JR on the Expression of VEGF and IL-8 under Hypoxic Condition

**Figure 4:**
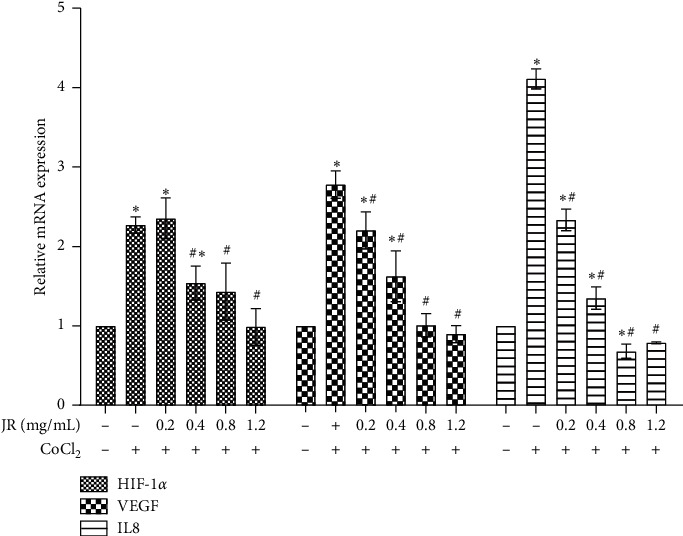
Effect of JR on the mRNA expression of VEGF and IL-8 under hypoxic condition. Huh 7 cells were treated with CoCl_2_ and JR for 24 h, and mRNA expression of HIF-1*α*, VEGF, and IL-8 was detected. Data are expressed as means ± S.D. and ^*∗*^*P* < 0.05 vs. control group and ^#^*P* < 0.05 vs. CoCl_2_ group.

Since the expression of VEGF and IL-8 is upregulated in the tumor hypoxic microenviroment, we then detected the transcriptional levels of VEGF and IL-8 in HCC cells under hypoxic condition. CoCl_2_ treatment significantly upregulated the expression of VEGF and IL-8 mRNA. Treatment with different concentrations of JR significantly inhibited the levels of VEGF and IL-8 mRNA in a dose-dependent manner (Figure 4).

**Figure 5:**
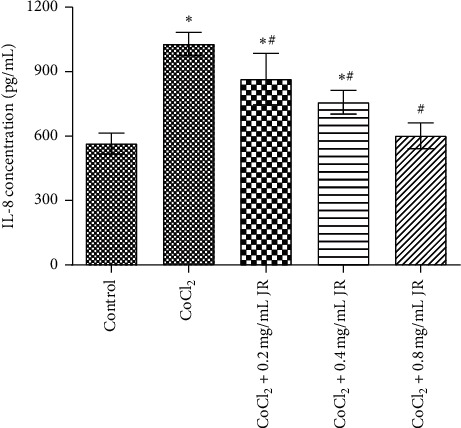
Effect of JR on the IL-8 protein secretion under hypoxic condition. Huh 7 cells were treated with CoCl_2_ and JR for 24 h, and ELISA assay was used to determine the IL-8 proteins in the culture media. Data are expressed as means ± S.D. and ^*∗*^*P* < 0.05 vs. control group and ^#^*P* < 0.05 vs. CoCl_2_ group.

We further confirmed the changes of IL-8 by ELISA. Under hypoxic condition, the concentration IL-8 significantly increased in the supernatant of Huh 7 cells. It was also antagonized by JR in a dose-dependent way (*P* < 0.05) (Figure 5).

### 3.6. JR Inhibited IL-8-Induced Angiogenesis through PI3K/AKT and MAPK/ERK Signals

**Figure 6:**
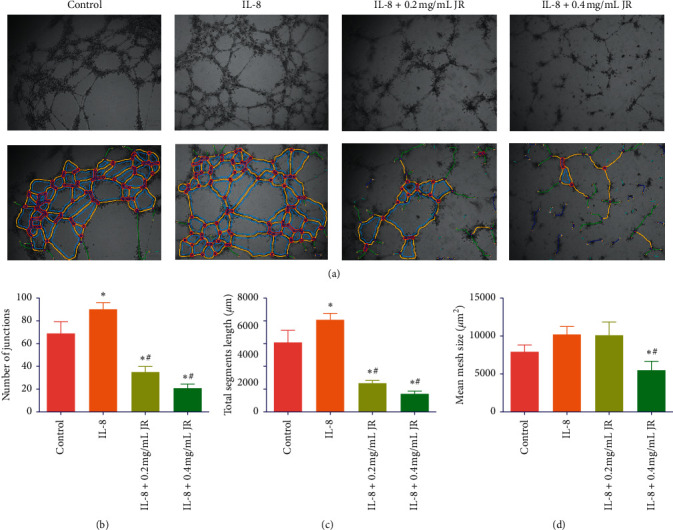
Effect of JR on IL-8-induced capillary-like tube formation. EA.hy 926 cells were treated with human IL-8 protein and JR for 16 h. (a) Inverted phase-contrast microscopy images of capillary-like tube formation and tubule branches marked out by Image J software (magnification of 100x). ((b)–(d)) Number of junctions, total segments length, and mean mesh size of the tube, respectively. Data are expressed as means ± S.D. ^*∗*^*P* < 0.05 vs. control group and ^#^*P* < 0.05 vs. IL-8 group.

It has been reported that IL-8 is able to induce the expression of VEGF independently [13] and the coexpression of IL-8 and HIF-1*α* is associated with metastasis and poor prognosis in HCC [23]. Therefore, we then observed whether IL-8 could promote the tube formation directly. IL-8 stimulation significantly promoted the tube formation of EA.hy 926 cells, which was suppressed by both 0.2 and 0.4 mg/mL of JR (Figure 6).

**Figure 7:**
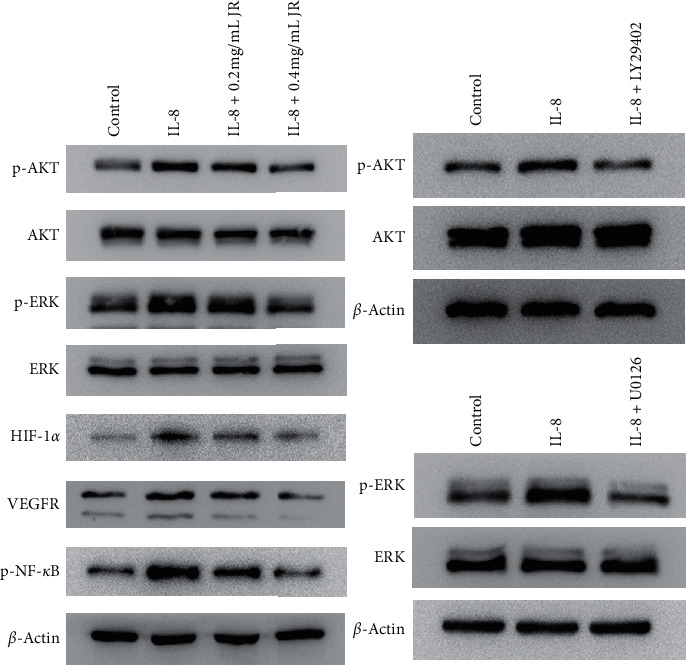
Effect of JR on p-AKT and p-ERK expression induced by IL-8. Western blot assay was used to detect the p-AKT, p-ERK p-NF-*κ*B, and VEGFR, the expression of p-AKT after treated with human IL-8 protein and PI3K inhibitor LY294002, and the expression of p-ERK after treated with human IL-8 protein and ERK inhibitor U0126. *β*-Actin served as the loading control.

Western blot showed that IL-8 upregulated the phosphorylation of AKT, ERK, and NF-*κ*B, which was inhibited by JR. JR may also inhibit the IL-8-induced upregulated VEGFR. On the other hand, effects of IL-8 on the p-AKT and p-ERK were also demonstrated by the inhibitor LY294002 and U0126, respectively (Figure 7).

## 4. Discussion

TACE is currently recognized as the preferred therapy for the unresectable liver cancer and provides the modest survival benefit [24]. The hypoxic microenvironment plays an important role in the tumor cell survival and metastasis and rapid formation of blood vessels after TACE. In addition, hypoxia-induced inflammatory cytokines, such as IL-8, stimulate angiogenesis, which may be an independent risk factor on HCC progression, metastasis, and recurrence [25–27]. In this study, we showed that JR may inhibit hypoxia-induced angiogenesis through suppressing IL-8/HIF-1*α*/PI3K and MAPK/ERK pathways after TACE in HCC patients.

TACE delivers lipiodol and chemotherapeutic drugs to obstruct tumor-feeding arteries and, thereby, induces a hypoxic microenvironment [28]. In the present study, CoCl_2_ was used to induce hypoxia-like condition and stimulated the formation of capillary-like tube, which was attenuated by JR treatment without inhibiting the proliferation of EA.hy 926 cells. HIF-1*α* is a heterodimer protein comprising an oxygen-sensitive *α* subunit and plays a critical role in regulating cellular responses to hypoxia and promotes angiogenesis by activating the expression of downstream-related genes such as VEGF [29, 30]. Our results also indicate that CoCl_2_ may upregulate the expression of HIF-1*α* and VEGF. By treating with different concentrations of JR, the levels of HIF-1*α* and VEGF were significantly inhibited in a dose-dependent way upon the hypoxic condition.

The response rates to TACE are heterogeneous since many factors may affect the prognosis of the disease [31]. Kim et al. [32] showed that IL-6 and IL-22 increase early after TACE. In our study, we detected the levels of several cytokines, including IL-1*β*, IL-2R, IL-6, and IL-8, which were all increased after TACE. PLR and NLR, which may represent convenient, quick, and noninvasive biological markers for the prognostic prediction in HCC [33–35], were also studied in this study. Interestingly, JR may downregulate the PLR in the early stage but in the late stage for NLR after TACE. It seems that JR performs a better role in PLR. Several reports have indicated that high IL-8 level correlates with reduced overall survival and may be served as a prognostic index independent of the target lesions' size or the patients' liver function or age [36, 37]. In this study, we found the JR suppressed the elevated IL-8 levels after TACE. These results indicate that JR may improve the prognosis of HCC patients through inhibiting hypoxia-induced angiogenesis in the HCC microenvironment.

IL-8 may predict poor prognosis in HCC when coexpressed with HIF-1*α* [23, 38]. In another study, the relationship of HIF-1*α* and IL-8 was evidenced by the downregulation of IL-8 in response to silencing of HIF-1*α* in HCC cell lines under hypoxic condition [39]. IL-8 knockdown inhibits angiogenesis and tumor growth in HCC independent of HIF-1*α* [40]. In our study, JR inhibited IL-8 upregulation under hypoxic condition and IL-8-induced capillary-like tube formation. Furthermore, phosphorylation of AKT, ERK, and NF-*κ*B was closely related to the tumor metastasis and angiogenesis [41], which was demonstrated in our study that IL-8 may stimulate the upregulation of p-AKT, p-ERK, and p-NF-*κ*B, which were inhibited by both JR. All our abovementioned results suggest that JR may inhibit the angiogenesis upon hypoxic condition through IL-8/HIF-1*α*/PI3K and MAPK/ERK pathways. Further study is needed to provide a more exact mechanism of the effects of JR and promote its clinical application.

## 5. Conclusions

In conclusion, JR can reduce the expression of many inflammatory cytokines in HCC patients after TACE. JR inhibits hypoxia-induced angiogenesis through suppressing IL-8/HIF-1*α*/PI3K and MAPK/ERK pathways after TACE in HCC patients. Also, further study is needed to provide more exact mechanism of the effects of JR and confirm its clinical efficacy.

## Figures and Tables

**Table 1:** Baseline of enrolled patients.

Variable	JR		Control		Total		*Z* value	*P* value
	*N*	Percent	*N*	Percent	*N*	Percent		
Age (years)							1.40	0.16
<55	14	34.1	23	48.9	37	42		
≥55	27	65.9	24	51.1	51	58		

Gender							0.99	0.33
Male	37	90.2	39	83	76	86.4		
Female	4	9.8	8	17	12	13.6		

Child–Pugh score							0.85	0.40
A	31	75.6	39	83	70	79.5		
B	10	24.4	8	17	18	20.5		

Performance status							0.47	0.64
Score 0	40	97.6	45	95.7	85	96.6		
Score 1	1	2.4	2	4.3	3	3.4		

BCLC stages							1.51	0.13
Stage A	6	14.6	5	10.6	11	12.5		
Stage B	25	61	23	48.9	48	54.5		
Stage C	10	24.4	19	40.4	29	33		

## Data Availability

The data used to support the findings of this study are available from the corresponding author upon request.
